# The Expanded Theory of Planned Behavior in the Context of Environmental Protection Behaviors for Undergraduates: Roles of Moral Norms and University Class Standings

**DOI:** 10.3390/ijerph19159256

**Published:** 2022-07-28

**Authors:** Angela Yi Jing Tsai, Alex Yong Kwang Tan

**Affiliations:** 1 Center of General Education, Tzu Chi University, Hualien 97004, Taiwan; tsai001@gms.tcu.edu.tw; 2 International College, Tzu Chi University, Hualien 97004, Taiwan

**Keywords:** theory of planned behavior (TPB), environmental protection behaviors, undergraduates, moral norms, class standings, structural equation modelling, questionnaire survey

## Abstract

The expanded Theory of Planned Behavior (ETPB) was applied to examine undergraduates’ environmental protection behaviors. Moral norms were applied into the model as the predictor of attitude, social norms and perceived behavioral control. The effects of different class standings were also examined. A questionnaire survey was conducted and 380 responses underwent data analysis using structural equation modelling. According to Model ETPB, perceived behavioral control and subjective norms were strongly affected by moral norms, while attitude was moderately affected by moral norms. Environmental protection behaviors was moderately affected by environmental protection intention, while environmental protection intention was moderately affected by perceived behavioral control which was the strongest predictor, followed by attitude and subjective norms. Invoking moral emotions through posters or peers leading by examples, which over time might internalize into moral norms, played an important role in positively affecting perceived behavioral control and subjective norms. This could be followed by simple and convenient programs creating a positive self-perception of the abilities to carry out environmental protection behaviors. When separated by class standings, perceived behavioral control was the strongest predictor for the freshmen class, while subjective norms were non-significant. For the class standing of sophomores and above, attitude was the strongest predictor.

## 1. Introduction

Many researches had conducted surveys regarding environmental protection, to understand the perceptions of different groups from various regions around the world. The targeted respondents included citizens from North America [1,2], Asia [3,4,5,6,7,8,9], Europe [10] and the Middle East [11,12], as well as students from North America [13,14], Asia [15,16,17,18,19], Europe [20,21,22] and the Middles East [23,24,25,26,27]. 

In addition, behavioral theories were frequently employed to explore the reasons behind various environmental protection behaviors. From the Theory of Reasoned Action [28] and the Theory of Planned Behavior [29], behavioral theories predicted that a person’s behavior was determined by their behavioral intention, which in turn was predicted by their attitude, subjective norms and perceived behavioral control. Since the development of the above theories, they had been applied to predict and explain various behaviors, including environmental related behaviors [30,31,32,33,34,35,36]. In recent years, together with the inclusion of other variables like environmental concerns [37,38,39], ethical values [40,41], habits [42,43], collectivism [44], innovation [45], altruism [46], responsibility [47] and motivation [48], various versions of the expanded Theory of Planned Behavior were actively applied in the field of environmental protection. 

Among various expanded Theory of Planned Behavior variables, “moral norms” was the most popular variable commonly inserted [36]. Defined as “the reflection of a personal value system in a given situation” [49], moral norms was widely applied together with the Theory of Planned Behavior in areas of environmental protection, with topics ranging from energy or electricity saving [50,51,52,53] to waste recycling [54,55,56,57,58,59]. Most literature inserted moral norms as the fourth variable beside attitude, social norms and perceived behavioral control, which positively affected behavioral intention [53,57,59]. Selected literatures attempted to replace attitude with moral norms [56], inserted moral norms as a predictor of attitude [58], inserted moral norms as a predictor of both attitude and behavioral intention [54], or inserted moral norms as a predictor of social norms [50]. 

As seen from growing literatures, the role of moral norms within the expanded Theory of Planned Behavior in the field of environmental protection was actively researched. The first focus of this research aimed to explore the expanded Theory of Planned Behavior where moral norms was the predictor of all three variables (attitude, social norms and perceived behavioral control) which positively affected behavioral intention, an unique and original attempt to extend the current field of research. This model of the expanded Theory of Planned Behavior was employed in the field of business ethics [60] and sexual risk behaviors [61] where moral principles were more important than adherence to rules and regulations, situations similar to the field of environmental protection. 

This model of the expanded Theory of Planned Behavior was tested among undergraduates studying in Tzu Chi University, Taiwan, similar to published literature where targeted respondents were students from a particular university in the United States [62], Australia [63], Hong Kong [64] or Lithuania [56]. By understanding the effects of various predictors from the expanded Theory of Planned Behavior for undergraduates from various regions of the world, cross-cultural comparison could be further examined [47,65]. 

In addition, the roles of demographic predictors within the Theory of Planned Behaviors were examined in various literatures [54], concentrating on the influences of gender [52], age [33,42], or educational level [33]. The second focus of this research aimed to explore the effect of university class standings on the environmental protection behaviors of undergraduates. This demographic predictor was hardly examined within the Theory of Planned Behavior but was showed to be influential according to published surveyed results regarding environmental protection and sustainability [66,67]. 

## 2. Materials and Methods

### 2.1. Research Hypothesis

From literature reviewed, most research focused on understanding the general awareness or attitude toward environmental protection around the world, targeting mostly residents, students or undergraduates. There were limited attempts to understand the factors influencing environmental protection behaviors of Taiwanese undergraduates and the consequences of their actions. 

**Figure 1:**
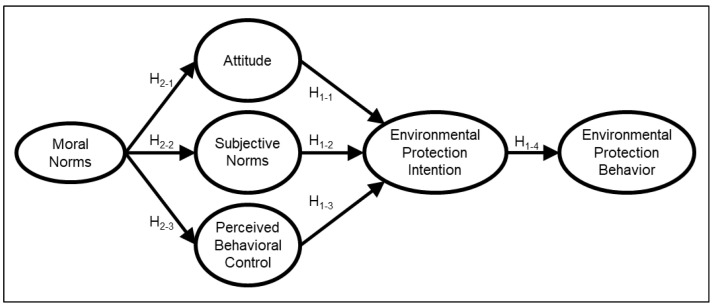
Theory of Planned Behavior and moral norms (Model ETPB) applied to environmental protection behaviors for undergraduates.

With references to the application of moral norms and the Theory of Planned Behavior for various topics [52,60,61], the following hypotheses (Figure 1) under the context of environmental protection behaviors for undergraduates at Tzu Chi University were proposed:

**Hypothesis** **1** **(H1).** *Moral norms positively affects attitude, subjective norms and perceived behavioral control regarding environmental protection behaviors for undergraduates (Model ETPB).*

**Hypothesis** **2** **(H2).** *Attitude, subjective norms and perceived behavioral control positively affect environmental protection intention, which in turn positively affects environmental protection behaviors for undergraduates (Model TPB).*

Furthermore, to understand the effects of environmental education, the collected data was divided into “freshmen only”, who started studying at the University for less than a month (Model A), as well as “sophomores and above”, who had studied at the University for more than a year (Model B).

### 2.2. Questionnaire Survey and Data Collection

A questionnaire survey was designed to collect data for testing the hypotheses. The survey was performed online, where informed consent was obtained from individuals before participating in the survey. SurveyCake, a frequently used online survey tool in Taiwan that employed cloud infrastructure services in compliance with general data protection regulations (GDPR) [68], had enabled “required questions” to ensure full completion of the survey, with no missing data and was familiar to local undergraduates. The survey underwent a pre-test of 20 personnel to assess its validity and identify unresolved ambiguities, with modifications that were made based on feedback obtained. This survey was widely distributed among the undergraduates from Tzu Chi University using an instant communications app and social media, from August to October 2021.

There were a total of roughly 3,300 undergraduates in Tzu Chi University [69] and in total 380 completed surveys were collected, fulfilling the 5.0% margin of error at 95% confidence level, using Equation (1)

(1) $$n=\frac{z^{2}p\left(1-p\right)N}{e^{2}N+z^{2}p\left(1-p\right)}$$

where *n* is the sample size, *N* is the population size, *e* is the margin of error, *z* is *z*-score according to desired confidence level, while *p* is the population proportion related to the survey and is usually given a value of 0.5. The sample size also satisfied the *n/q* rule of the analytic method of structural equation modelling having a value within 5 to 20 [70], where *n* is the sample size and *q* is the number of parameters to be estimated. Furthermore, according to the central limit theorem, the assumption of normality matters less for large sample size, where an average of 100 to 160 sample sizes were required for heavy-tailed distributions, according to published literature [71].

The survey used in this study consisted of two sections, with a total of 25 questions. The first section had a total of three questions, collecting basic demographic data (as shown in Table 1) which included gender, age and class standings. The second section had a total of 22 questions for six constructs, regarding moral norms, attitude, subjective norms, perceived behavioral control, environmental protection intention and environmental protection behaviors of respondents.

### 2.3. Measures 

Each construct was measured by three to five items, rated based on the five-point Likert scale, where 1 indicated “strongly disagree” or “never” and 5 indicated “strongly agree” or “always.” Measures of awareness, subjective norms, perceived behavioral control and behavioral intentions were modified from previous studies, while measures of moral norms were adopted based on previous studies. The environmental behavior questions were created after discussion with personnel involved with environment protection policies and were widely encouraged behaviors among undergraduates. 

The moral norms construct consisted of four items. The first item “I am confident that my actions uphold moral values” and the second item “It is important that my friends and family uphold moral values,” allowed respondents to reflect upon their moral values, which were adopted and modified from published literature [72,73]. The third item “Environmental protection is a moral issue” and the fourth item “Through environmental protection, I find additional meaning in life,” were adopted and modified from existing literature [53,55,57,64], where personnel involved with the pre-test felt that “moral issue” was a better reflection of Taiwanese society, compared to “moral obligation.” Similarly, “guiltiness” was replaced by “additional meaning in life” as positive psychology is widely incorporated in Taiwan contemporary education.

The attitude construct was comprised of three items, modified from contemporary researches [31,40,44] where the first item “I believe that environmental protection is very important” and the second item “I will protect our Earth’s environment,” tested the strength of respondents’ behavioral beliefs. Respondents from the pre-test reflected that “very important” was a better choice of wordings, compared to “valuable” or “beneficial,” as it was more straightforward. The third item “Environmental protection gives plants a better environment in which to grow” aimed to understand respondents’ attitudes and respect for nature; another item regarding animals “Environmental protection saves polar bears from possible extinction” modified from literature [74], was viewed as disconnected from Taiwanese society by respondents from the pre-test and hence removed. 

The social norms construct was made up of three items, modified from previous researches [40,44,64], where respondents from the pre-test believed that “friends and family” was a better phrase in Taiwanese society compared to “people who are important to me.” The first item “My friends and family are concerned about environmental protection” tested the respondents’ identification with a referent, while the second item “My friends and family supported me in concerning about environmental protection,” and the third item “My friends and family supported me in adopting environmental protection behaviors” tested respondents’ motivations to comply.

The perceived behavioral control construct consisted of three items and were modified from existing researches [44,64]. The first item “I am confident that if I want, I can protect the environment” tested the respondents’ powers of control. The second item “I have sufficient time to protect the environment” and the third item “I have limitless potential in protecting the environment” tested the strength of control belief, where “limitless potential” replaced “opportunities.”

The environmental protection intention construct included four items from published researches [44,64]. The first item “I intend to protect the environment” and the second item “I am glad to adopt environmental protection behaviors” attempted to understand respondents’ general behavioral intention. The third item “I am willing to use my money to protect the environment” and the fourth item “I am willing to use my time to protect the environment” were a single item in literature [44], but was advised by respondents from the pre-test to separate into two items as Taiwanese view money and time differently. 

Items from the environmental protection behaviors construct were created after discussion with personnel involved with environment protection policies and followed existing measures, where behavioral intention was general in nature, while behaviors were with respect to specific environmental protection behaviors [64]. The first item “I switch off lights and other electric appliances when not in use” was similar to published literature [64] while the rest of the items “I take the stairs instead of using the elevator when walking up/down less than three floors”; “I use reusable bags instead of disposable bags”; “I sort my rubbish according to regulations”; and “I use reusable eating utensils instead of disposable eating utensils” were widely promoted and encouraged behaviors among undergraduates.

### 2.4. Statistical Processing 

To analyze the collected data, descriptive statistics and structural equation modelling were carried out. Structural equation modelling was a statistical methodology to establish “causal” relationships among variables, allowing a clear conceptualization of the impact of social psychological factors, using the expanded Theory of Planned Behavior on environmental protection behaviors of undergraduates. 

The two major software tools used were SPSS 25.0 and Amos 20.0. Firstly, the internal consistency reliability and construct validity were examined. Secondly, a confirmatory factor analysis model was analyzed in order to determine an adequate measurement model. Models were estimated by the Maximum Likelihood method, covariances were estimated among all exogenous variables and control variables, as well as tested with the bootstrap approach. The overall model fit was measured by standardized root mean square residual (SRMR ≤ 0.08), the root mean square error of approximation (RMSEA ≤ 0.08), the goodness of fit (GFI ≥ 0.85), the adjusted goodness of fit (AGFI ≥ 0.80), Parsimony goodness of fit (PGFI ≥ 0.50) and the comparative fit index (CFI ≥ 0.90) [75,76,77,78]. 

## 3. Results 

### 3.1. Statistical Fitness 

From Table 2, all values of skewness (maximum absolute value of 1.087) and kurtosis (maximum absolute value of 1.987) were well within the normality criteria of between −3.0 and +3.0 as well as −10.0 and +10.0, respectively [78]. The structural equation modelling reported good overall fit to the data (Model ETPB) according to Table 3. Values of fit statistics for the confirmatory factor analysis model were all within the desirable ranges, where SRMR = 0.049; RMSEA = 0.076; GFI = 0.873; AGFI = 0.830; PGFI = 0.652; CFI = 0.910. 

A good measurement technique had to be both reliable and valid. Cronbach’s alpha above 0.700 shows adequate reliability [78] and all constructs in this present study fulfilled this criterion, showing moderate or high internal consistency reliability, as shown in Table 4. In the confirmatory factor analysis model, each construct was well qualified for structural regression models, as each construct has at least three measures [78]. The Kaiser-Meyer-Olkin test produced values greater than 0.700 for all constructs and each construct had only a single eigenvalue with value greater than 1, indicating good construct validity from exploratory factor analysis [78]. Furthermore, all measures were significantly associated with the specified constructs (*p* < 0.001) with standardized loadings larger than 0.500, as seen in Table 5. 

The standardized regression coefficients for Model ETPB are shown in Table 6. According to the model fit statistics, the SEM fits the data well, as shown in Table 3, with SRMR = 0.050; RMSEA = 0.076; GFI = 0.871; AGFI = 0.832; PGFI = 0.668; CFI = 0.910. 

### 3.2. Structural Relationships 

**Figure 2:**
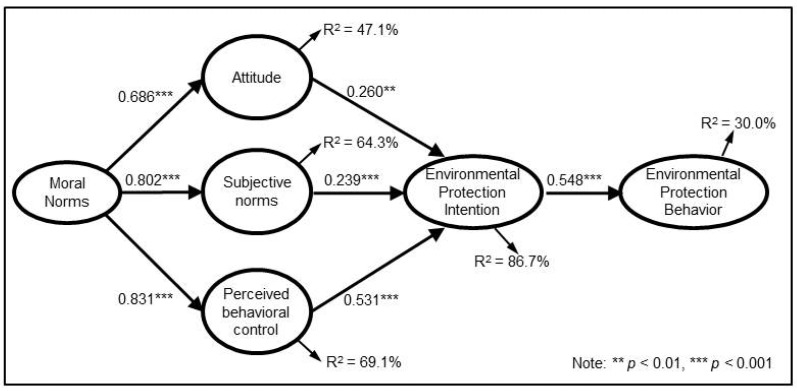
Standard estimates for expanded Theory of Planned Behavior (Model ETPB).

The standardized estimates for Model ETPB are elaborated in Figure 2 and Table 7. Moral norms had a significantly positive correlation with attitude (β = 0.686, *p* < 0.001), subjective norms (β = 0.802, *p* < 0.001) and perceived behavioral control (β = 0.831, *p* < 0.001). In addition, 47.1%, 64.3% and 69.1% of the variance in undergraduates’ attitudes, subjective norms and perceived behavioral control, respectively were accounted for by moral norms. Attitude (β = 0.260, *p* < 0.01), subjective norms (β = 0.239, *p* < 0.001) and perceived behavioral control (β = 0.531, *p* < 0.001) were positively related to the intention of environmental protection behaviors. Environmental protection intention (β = 0.548, *p* < 0.001) had a significantly positive relationship with environmental protection behaviors. At the same time, 86.7% of the variance in undergraduates’ environmental protection intention and 30.0% of the variance in their environmental protection behaviors were accounted for.

Considering the original Theory of Planned Behavior, attitude (β = 0.292, *p* < 0.001), subjective norms (β = 0.218, *p* < 0.01) and perceived behavioral control (β = 0.539, *p* < 0.001) were positively related to the intention of environmental protection behaviors. Environmental protection intention (β = 0.545, *p* < 0.001) had a significantly positive relationship with environmental protection behaviors. Furthermore, 88.3% of the variance in undergraduates’ environmental protection intention and 29.7% of the variance in their environmental protection behaviors were accounted for. 

For the freshmen only class standing (Model A), attitude (β = 0.249, *p* < 0.001) and perceived behavioral control (β = 0.726, *p* < 0.001) were positively related to the intention of environmental protection behaviors. However, subjective norms was not a significant predictor of environmental protection intention. Environmental protection intention (β = 0.490, *p* < 0.001) had a significantly positive relationship with environmental protection behaviors. At the same time, 90.1% of the variance in undergraduates’ environmental protection intention and 24.0% of the variance in their environmental protection behaviors were accounted for. For the class standings of sophomores and above (Model B), attitude (β = 0.414, *p* < 0.001), subjective norms (β = 0.306, *p* < 0.01) and perceived behavioral control (β = 0.326, *p* < 0.01) were positively related to the intentions of environmental protection behaviors. Environmental protection intention (β = 0.558, *p* < 0.001) had a significantly positive relationship with environmental protection behaviors. At the same time, 85.7% of the variance in undergraduates’ environmental protection intention and 31.1% of the variance in their environmental protection behaviors were accounted for.

## 4. Discussion 

### 4.1. Effects of Moral Norms According to Model ETPB 

Botetzagias et al. [51] examined the electricity saving behaviors of Greek households using the expanded Theory of Planned Behavior of moral norms, age and gender, while Chan and Bishop [63] conducted surveys to understand the recycling behaviors of residents in Western Australia. Results from the above literatures showed that moral concerns are predominately interwoven within the attitude, subjective norms and perceived behavioral control constructs, whereas moral norms were found to be moderately correlated to attitude, as well as weakly correlated to subjective norms and perceived behavioral control.

Although most literature fitted moral norms as the fourth predictor of behavioral intention within the expanded Theory of Planned Behavior, there were growing researches that considered the effects of moral norms on behavioral intention being mediated by the original constructs of the Theory of Planned Behavior [49]. Bhutto et al. [50] showed that moral norms was not a significant predictor of social norms in Pakistani residents’ decisions to purchase energy-efficient appliances. Botetzagias et al. [54] attempted to understand the recycling behaviors of residents in Greece and found moral norms to be a strong and significant predictor of attitude. Similarly, Zhang et al. [58] explored the behavior of waste sorting by residents in Guangzhou, China and determined that moral norms was a strong and significant predictor of attitude. 

Current results showed that moral norms strongly predicted the effects of perceived behavioral control and subjective norms, where social pressures and positive self-perception were still greatly influenced by the moral values upheld by Taiwanese young adults. The acceptance of positive moral emotions like empathy and gratitude within the Taiwanese community, was widely applied to environmental protection where “empathizing with the destruction of Mother Earth” or “giving gratitude towards the environment for providing us the resources we need” were recognized moral standards, providing the motivating forces for conforming with social pressures and the perceived control of doing good [79]. The prediction of attitude by moral norms was significant, but relatively weaker, as attitude was predominately influenced by knowledge rather than moral values, after years of incorporating environmental education within Taiwan’s twelve years of compulsory education. These differences in findings compared with published literatures [54,58] could be due to generational differences in environmental education among the various targeted respondents, as well as the social cohesiveness of Taiwanese society. These findings showed that invoking moral emotions, which internalized into moral norms played an important role in positively affecting subjective norms and perceived behavioral control, which in turn positively affects behavioral intention. For instance, posters around the elevator lobby showcasing the amount of carbon emission saved when stairs were taken instead of using the elevator, or cashiers saying “Thank you for saving the environment” when reusable bags were used, appealed to the moral emotions, which over time might internalize into moral norms. 

García Mejías et al. [31] examined factors influencing the intention to take up environmental protection behaviors by tourist operators. Results listed perceived behavioral control as the most significant predictor, followed by subjective norms. However, attitude was found to be statistically insignificant. Nie et al. [33] investigated the factors influencing careful usage of energy saving behaviors among residents in Changchun, China. Results showed that subjective norms were the most significant factor affecting residents’ intentions to save energy. Surveyed from non-academic employees from a Canadian University, Yuriev et al. [36] found that attitude, followed by perceived behavioral control, were the strongest predictor of behavioral intention regarding environmental protection behaviors in the workplace. Swaim et al. [62] explored the environmental protection behaviors of university undergraduates from the United States, showing that attitude was the strongest predictor of behavioral intention, followed by subjective norms. Lin [74] targeted residents from Kaohsiung, Taiwan and found that perceived behavioral control was the most significant predictor affecting environmental protection behaviors.

The standard estimates showed perceived behavioral control as the strongest predictor, which was similar to García Mejías et al. [31] and Lin [74] but different from Nie et al. [33], Yuriev et al. [36] and Swaim et al. [62]. Discussions with undergraduates from revealed that implementation of environmental education since elementary schools equipped students with a strong and positive attitude of environmental protection (mean of 4.47 according to Table 4), which did not translate into either environmental protection intention or environmental protection behaviors, a phenomenon known as the debunked “knowledge-attitude-behavior theory,” which were widely observed [80]. This weak showing of attitude as a predictor of behavioral intention was similarly observed in Lin [74], where the survey was also conducted in Taiwan and more than half of its respondents were young adults. Meanwhile, the various environmental protection behaviors were relatively personal decisions that undergraduates might make regardless of social pressure, weakening the effect of subjective norms. Hence, the role of perceived behavioral control was highlighted, where respondents viewed environmental protection behaviors as actions without significant constraints. These findings showed that a positive self-perception of the abilities to carry out environmental protection behaviors played an important role, where future campaigns or programs targeting environmental protection behaviors should be kept simple and convenient, allowing participants to connect to the heart of the matter easily. For example, including reusable chopsticks (size of a large pen) as part of the orientation package for freshmen upon enrollment, allowed undergraduates to experience the convenience of carrying around reusable chopsticks and further increased their positive self-perception of the ability to exhibit environmental protection behaviors.

### 4.2. Effects of Class Standings 

Tzu Chi University, a recipient of the National Sustainable Development (Educational Institutions) Awards [81], incorporated strict and comprehensive environmental education programs, which were compulsory for all freshmen, including lessons and hands-on training. The dormitory recycling system consisted of 11 classifications (aluminum cans, iron cans, PET bottles, PET bottle caps, general plastic, paper, carton boxes, paper takeout boxes, batteries, kitchen waste and general waste) and energy saving behaviors were widely encouraged. Hence, comparison for different class standings yielded several interesting results.

The freshmen class standing consisted of undergraduates, who enrolled in Tzu Chi University for less than a month. Deep bonding of friendships was yet to be formed and the understandings of university’s environmental protection policies were still at the infancy stage, accounting for the non-significant predictor of subjective norms toward environmental protection intention. Students reflected that through social media, they heard various versions of the University’s environmental protection policies and received many assurances from undergraduates of higher class standings, giving a relatively higher self-perception of the abilities to perform environmental protection behaviors. After studying at Tzu Chi University for more than a year and exposure to the various environmental education programs, the class standings of sophomores and above had a greater understanding and knowledge of environmental protection, allowing attitude to become a stronger predictor of environmental protection intention. Furthermore, deeper involvements with the university’s environmental protection policies lowered their perceived behavioral control as a predictor of environmental protection intention, as a more realistic self-perception of the abilities to perform environmental protection behaviors was formed. Lastly, the formation of friendships deepened social pressures, and the subjective norms became a significant predictor of environmental protection intention. These findings showed that environmental education should be continued within tertiary education, in order to maintain the positive effects of attitude on behavioral intention. Enlisting freshmen as environmental protection ambassadors to lead and drive environmental protection behaviors among fellow freshmen might help to strengthen social norms. Furthermore, these ambassadors could lead by example, showing that environmental protection behaviors are simple and convenient, in order to slow down the weakening of perceived behavioral control.

## 5. Conclusions

This research applied the expanded Theory of Planned Behavior as a theoretical model and conducted a questionnaire survey to obtain empirical results from undergraduates at Tzu Chi University. Next, the obtained results underwent verification through data analysis using structural equation modelling. Analyzed results from the expanded Theory of Planned Behavior (Model ETPB) showed that perceived behavioral control and subjective norms were strongly affected by moral norms while attitude was moderately affected by moral norms, thus accepting Hypothesis 1. Analyzed results from both Model ETPB and Model TPB showed that environmental protection behaviors was moderately affected by environmental protection intention, while environmental protection intention was moderately affected by perceived behavioral control which was the strongest predictor, followed by attitude and subjective norms. Hence, Hypothesis 2 was accepted. 

Analyzed results according to class standings showed that after undergoing the strict and compulsory environmental education programs conducted by Tzu Chi University, attitude became a better predictor of environmental protection intention due to greater environmental knowledge. In addition, the formation of friendships and a better understanding of the university’s environmental protection policies increased the societal pressure of environmental protection, improving the prediction of environmental protection intention by subjective norms from non-significant for freshmen only (Model A) to weakly affected for sophomores and above (Model B). Finally, after a more realistic self-perception of the abilities to perform environmental protection behaviors was formed during the higher years of class standings (Sophomore and above), the prediction of environmental protection intention by perceived behavioral control dropped from strongly affected for freshmen only (Model A) to weakly affected for sophomores and above (Model B).

Energy saving and environmental protection interventions in university should be selected carefully according to validated behaviors and behavior change theories. Appropriate intervention approaches invoking moral emotions which over time might internalize into moral norms, such as faculties and selected students leading by example in improving their environmental protection behaviors could strengthen subjective norms as a predictor of environmental protection intention. Furthermore, education and information campaigns or posters highlighting individual’s ease and control in environmental protection could shape and enhance environmental protection intention by improving perceived behavioral control. In addition, stronger efforts were required to improve environmental protection intention as the predictor of environmental protection behaviors. Future researches could concentrate on examining the effects of university’s environmental education programs on its undergraduates, in order to better predict and improve their environmental protection behaviors.

## Figures and Tables

**Table 1:** Characteristics of surveyed sample.

Variables	*n* = 380	Percentage
**Gender**		
Male	107	28.2%
Female	266	70.0%
Declined to disclose	7	1.8%
**Age**		
18	111	29.2%
19	127	33.4%
20	54	14.2%
21	47	12.4%
22	21	5.5%
>22	20	5.3%
**Class Standings**		
Freshmen	225	59.2%
Sophomores and above	155	40.8%

**Table 2:** List of constructs and their mean, standard deviation, skewness and kurtosis respectively.

Item	Question	Mean	S.D.	S.	K.
	**Moral Norms**				
MN1	I am confident that my actions uphold moral values.	4.17	0.718	−0.692	0.587
MN2	It is important that my friends and family uphold moral values.	4.03	0.808	−0.349	−0.525
MN3	Environmental protection is a moral issue.	4.11	0.870	−0.816	0.512
MN4	Through environmental protection, I find additional meaning in life.	3.68	0.943	−0.250	−0.166
	**Attitude**				
AT1	I believe that environmental protection is very important.	4.50	0.610	−1.087	1.987
AT2	I will protect our Earth’s environment.	4.38	0.645	−0.789	1.035
AT3	Environmental protection gives plants a better environment in which to grow.	4.52	0.574	−0.703	−0.500
	**Subjective** **Norms**				
SN1	My friends and family are concerned about environmental protection.	3.66	0.929	−0.267	−0.241
SN2	My friends and family supported me in concerning about environmental protection.	3.92	0.886	−0.508	0.083
SN3	My friends and family supported me in adopting environmental protection behaviors.	3.92	0.834	−0.317	−0.197
	**Perceived** **Behavioral** **Control**				
PC1	I am confident that if I want, I can protect the environment.	4.05	0.764	−0.697	1.069
PC2	I have sufficient time to protect the environment.	3.59	0.944	−0.323	−0.067
PC3	I have limitless potential in protecting the environment.	3.83	0.910	−0.605	0.337
	**Environmental** **Protection Intention**				
IN1	I intend to protect the environment.	4.19	0.708	−0.643	0.944
IN2	I am glad to adopt environmental protection behaviors.	4.07	0.775	−0.422	−0.055
IN3	I am willing to spend my money to protect the environment.	3.63	0.902	−0.325	0.133
IN4	I am willing to use my time to protect the environment.	3.96	0.781	−0.427	0.348
	**Environmental protection behaviors**				
EB1	I switch off lights and other electrical appliances when not in use.	3.89	0.960	−0.748	0.238
EB2	I take the stairs instead of using the elevator when walking up/down less than three floors.	3.55	1.142	−0.241	−0.961
EB3	I use reusable bags instead of disposable bags.	3.35	1.078	−0.141	−0.658
EB4	I sort my rubbish according to regulations.	4.13	0.902	−1.006	0.767
EB5	I use reusable eating utensils instead of disposable eating utensils.	3.94	0.931	−0.653	0.060

Note: S.D. refers to standard deviation, S. refers to skewness and K. refers to kurtosis.

**Table 3:** Goodness of fit for Model ETPB.

Parameters	Desirable Range	CFA	SEM
SRMR	≤0.08	0.049	0.050
RMSEA	≤0.08	0.077	0.076
GFI	≥0.85	0.873	0.871
AGFI	≥0.80	0.830	0.832
PGFI	≥0.50	0.652	0.668
CFI	≥0.90	0.910	0.910

**Table 4:** Summary of latent variables for Model ETPB.

Constructs	Measures	Cronbach’s Alpha	K.M.O. Values	Mean	S.D.
Moral Norms	MN1-4	0.747	0.747	4.00	0.859
Attitude	AT1-3	0.863	0.725	4.47	0.541
Subjective Norms	SN1-3	0.860	0.720	3.83	0.781
Perceived Behavioral Control	PC1-3	0.827	0.723	3.83	0.755
Intention	IN1-3	0.855	0.804	3.96	0.663
Environmental protection behaviors	EB1-4	0.718	0.787	3.77	0.690

Note: K.M.O. refers to Kaiser-Meyer-Olkin and S.D. refers to standard deviation.

**Table 5:** Results of confirmatory factor analysis for Model ETPB.

Constructs	Measures	Standardized Regression Weights
Moral Norms	MN1	0.740 ***
	MN2	0.724 ***
	MN3	0.582
	MN4	0.770 ***
Attitude	AT1	0.827
	AT2	0.871 ***
	AT3	0.707 ***
Subjective Norms	SN1	0.686
	SN2	0.784 ***
	SN3	0.886 ***
Perceived Behavioral Control	PC1	0.815
	PC2	0.757 ***
	PC3	0.793 ***
Intention	IN1	0.839
	IN2	0.800 ***
	IN3	0.660 ***
	IN4	0.854 ***
Environmental Protection Behavior	EB1	0.552
	EB2	0.509 ***
	EB3	0.696 ***
	EB4	0.536 ***
	EB5	0.628 ***

Note: regression weights of MN3, AT1, SN1, PC1, IN2 and EB1 were assumed to be 1.000; *** *p* < 0.001.

**Table 6:** SEM standardized regression coefficients for various models.

Constructs	Measures	ETPB	TPB	A	B
Moral Norms	MN1	0.741 ***	-	-	-
	MN2	0.723 ***	-	-	-
	MN3	0.584	-	-	-
	MN4	0.763 ***	-	-	-
Attitude	AT1	0.821	0.877	0.854	0.903
	AT2	0.877 ***	0.835 ***	0.860 ***	0.808 ***
	AT3	0.703 ***	0.760 ***	0.794 ***	0.719 ***
Subjective Norms	SN1	0.687	0.700	0.741	0.772
	SN2	0.785 ***	0.802 ***	0.836 ***	0.874 ***
	SN3	0.885 ***	0.867 ***	0.851 ***	0.808 ***
Perceived Behavioral Control	PC1	0.815	0.805	0.784	0.837
	PC2	0.755 ***	0.772 ***	0.778 ***	0.758 ***
	PC3	0.793 ***	0.790 ***	0.815 ***	0.758 ***
Intention	IN1	0.840	0.849	0.858	0.838
	IN2	0.795 ***	0.788 ***	0.759 ***	0.799 ***
	IN3	0.665 ***	0.619 ***	0.659 ***	0.613 ***
	IN4	0.859 ***	0.846 ***	0.847 ***	0.870 ***
Environmental Protection Behavior	EB1	0.554	0.555	0.575	0.495
	EB2	0.506 ***	0.508 ***	0.446 ***	0.520 ***
	EB3	0.701 ***	0.701 ***	0.784 ***	0.689 ***
	EB4	0.537 ***	0.537 ***	0.656 ***	0.512 ***
	EB5	0.620 ***	0.618 ***	0.564 ***	0.689 ***

Note: Regression weights of MN3, AT1, SN1, PC1, IN2 and EB1 were assumed to be 1.000; *** *p* < 0.001.

**Table 7:** Standard estimates for various models.

Standard Estimates	ETPB	TPB	A	B
AT ← MN	0.686 ***	-	-	-
SN ← MN	0.802 ***	-	-	-
PC ← MN	0.831 ***	-	-	-
IN ← AT	0.260 **	0.292 ***	0.249 ***	0.414 ***
IN ← SN	0.239 ***	0.216 **	0.062 (N.S.)	0.306 ***
IN ← PC	0.531 ***	0.539 ***	0.726 ***	0.326 **
EB ← IN	0.548 ***	0.545 ***	0.490 ***	0.558 ***
AT (R^2^)	0.471	-	-	-
SN (R^2^)	0.643	-	-	-
PC (R^2^)	0.691	-	-	-
IN (R^2^)	0.867	0.883	0.901	0.857
EB (R^2^)	0.300	0.297	0.240	0.311

Note: N.S. refers to non-significant; ** *p* < 0.01, *** *p* < 0.001.

## Data Availability

The dataset will be provided upon request.
